# The effects of augmented visual feedback during balance training in Parkinson’s disease: study design of a randomized clinical trial

**DOI:** 10.1186/1471-2377-13-137

**Published:** 2013-10-04

**Authors:** Maarten RC van den Heuvel, Erwin EH van Wegen, Cees JT de Goede, Ingrid AL Burgers-Bots, Peter J Beek, Andreas Daffertshofer, Gert Kwakkel

**Affiliations:** 1 MOVE Research Institute Amsterdam, Faculty of Human Movement Sciences, VU University Amsterdam, van der Boechorststraat 9, Amsterdam, 1081 BT, The Netherlands; 2 Department of Rehabilitation Medicine, MOVE Research Institute Amsterdam, VU University Medical Center, De Boelelaan 1118, Amsterdam, 1007 MB, The Netherlands

**Keywords:** Randomized clinical trial, Parkinson’s disease, Physical therapy, Balance training, Postural control, Virtual reality, Visual feedback, Electroencephalography, Posturography, Force plate

## Abstract

**Background:**

Patients with Parkinson’s disease often suffer from reduced mobility due to impaired postural control. Balance exercises form an integral part of rehabilitative therapy but the effectiveness of existing interventions is limited. Recent technological advances allow for providing enhanced visual feedback in the context of computer games, which provide an attractive alternative to conventional therapy. The objective of this randomized clinical trial is to investigate whether a training program capitalizing on virtual-reality-based visual feedback is more effective than an equally-dosed conventional training in improving standing balance performance in patients with Parkinson’s disease.

**Methods/design:**

Patients with idiopathic Parkinson’s disease will participate in a five-week balance training program comprising ten treatment sessions of 60 minutes each. Participants will be randomly allocated to (1) an experimental group that will receive balance training using augmented visual feedback, or (2) a control group that will receive balance training in accordance with current physical therapy guidelines for Parkinson’s disease patients. Training sessions consist of task-specific exercises that are organized as a series of workstations. Assessments will take place before training, at six weeks, and at twelve weeks follow-up. The functional reach test will serve as the primary outcome measure supplemented by comprehensive assessments of functional balance, posturography, and electroencephalography.

**Discussion:**

We hypothesize that balance training based on visual feedback will show greater improvements on standing balance performance than conventional balance training. In addition, we expect that learning new control strategies will be visible in the co-registered posturographic recordings but also through changes in functional connectivity.

**Trial registration:**

ISRCTN: ISRCTN47046299

## Background

Patients with Parkinson’s disease (PD) typically suffer from motor symptoms such as rigidity, tremor, bradykinesia, and postural instability, and are often confronted with serious mobility-related deficits such as problems with walking, balance, and transfers (e.g., standing up and sitting down, turning in bed). As the disease progresses, the patient’s functional capacity typically declines. This can severely impact activities of daily living [1], resulting in a downward spiral of immobility and deconditioning [2]. Impaired postural control is an important contributor to falls [3,4]. Physical therapy may positively affect mobility in PD, but exercises need to be task-specific and prescribed in sufficient dose [5-8]. In the case of impaired postural control this requires the inclusion of balance exercises in training programs [9].

A prerequisite for having patients fully involved and adhere to a training protocol is that the exercises are meaningful, engaging, and challenging. Novel technological developments allow for integrating a patient’s own movements in virtual environments, alongside other elements borrowed from the gaming industry, such as real-time 3D rendering, avatars, and score-keeping. Balance exercises are now often included in game consoles (such as Nintendo Wii™). Given the evidence that externally guided movements are mediated by neural pathways that differ from those involved in internally guided movements [10-12] and considering the extensive evidence regarding the benefits of using external stimuli in patients with PD [13-16] the provision of explicit, augmented visual feedback (VF) of a patient’s own movements in a virtual environment may be an important element in rehabilitation interventions in patients with PD.

To date, there are few reports on the additive value of VF to balance training paradigms for patients with PD. Esculier and co-workers [17] investigated the effects of home-based balance training using Nintendo Wii Fit and reported improvements in functional balance but a control group was not included. Pompeu and co-workers [18] did not find additional benefits of Nintendo Wii-based training compared to control therapy, but the contrast of the intervention between the groups was limited due to the fact that VF exercises only made up a portion of the therapy provided in the experimental group.

In the present randomized clinical trial (RCT) we will compare the effects of VF-based balance training on standing balance performance in patients with PD with the effects of conventional balance training. In addition, we will investigate to what degree the improvements in standing balance are accompanied by changes in cortical activity. To this end, we will employ whole-head electroencephalographic (EEG) recordings during a postural task [19,20].

We hypothesize that VF-based training is more effective than usual training in improving standing balance performance, with functional reach distance as the primary outcome. We also hypothesize that VF-related changes in balance performance are associated with more pronounced (movement-related changes in) beta synchronization in primary motor areas [21-23] and corresponding changes in functional connectivity of the entire motor network (including, e.g., pre-motor and supplementary motor areas), which may reflect the learning of novel strategies to control standing balance.

## Methods

### Design and procedures

**Figure 1:**
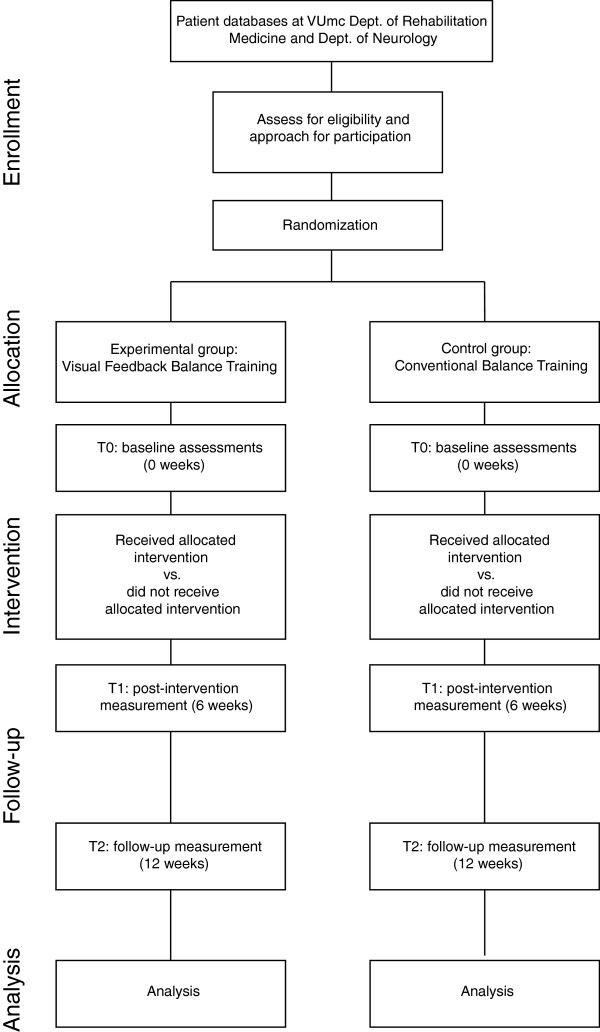
Flowchart of the trial.

This study is a pilot RCT comparing two treatment groups of patients with PD (Figure 1). Patients are allocated to either a five-week training program with balance exercises containing augmented VF (experimental group), or a five-week balance training program that follows existing guidelines (control group). Assessments will take place before intervention (T0), at six weeks (T1), and at twelve weeks (T2). Intervention assignment will be concealed by drawing opaque sealed envelopes by an independent investigator not involved in the study. A blinded researcher will perform the assessments. Patients will be asked to refrain from mentioning the nature of the intervention they receive to the researcher. All assessments will be performed in the ON-phase of levodopa medication. To minimize the effects of (changes in) PD medications on the patients’ physical performance, the time of day at which patients are assessed will be kept constant throughout the study.

The protocol has been approved by the Medical Ethics Committee of the VU University medical center (VUmc) Amsterdam and is registered with Current Controlled Trials under ISRCTN47046299.

### Participants

A convenience sample of 36 patients will be recruited from the Neurology Department and from patient databases of the Department of Rehabilitation Medicine, VUmc. Patients who are likely to meet the criteria for inclusion will be invited to participate in the RCT.

Inclusion criteria will be (i) a diagnosis of idiopathic PD, mild to moderate stage (i.e. Hoehn & Yahr stages II and III), (ii) able to participate in either of the training programs, and (iii) written and verbal informed consent. Exclusion criteria are the presence of neurological, orthopedic, or cardiopulmonary problems that can impair participation, insufficient cognitive function (Mini Mental State Examination, MMSE < 24), an unstable medication regime, and any condition that renders the patient unable to understand or adhere to the protocol such as cognitive, visual, and/or language problems.

### Intervention

The intervention will contain ten treatment sessions of 60 minutes each over a period of five weeks. In order to efficiently organize the training, different workstations related to standing balance will be organized in a circuit allowing six patients to train simultaneously. To capitalize on the benefits of action observation [24] patients will work in pairs at each workstation. Patients will take turns performing the exercise while the other person observes and/or rests. The training paradigm will be applied to both the experimental and the control group.

In the experimental group VF is explicitly integrated in each workstation (see below for details). In the control group workstations will consist of balance exercises that follow the current guidelines for physical therapy in PD [9]. The exercises in both groups will focus on controlling body posture in forward and sideways direction, exploring limits of stability, weight-shifting, sit-to-stand exercises, and dual-task exercises. Which specific workstation will be used for which treatment session will be decided before the start of the training program. Two expert therapists (CdG and IB) will define training goals, monitor the training intensity during the sessions, and keep time. Throughout the training program, the therapists will monitor individual progress and progressively adjust personal training goals. If desired, exercise complexity and/or workload will be increased throughout the sessions. All training sessions will take place in an outpatient setting of VUmc. All participants will be asked to keep a training and fall log during the duration of the training program. Interventions will be rated by the participants in terms of the perceived exertion over the entire training session.

The content of each treatment session will be controlled for type and duration as shown in Table 1. Warming-up and cooling-down exercises of about 5 minutes will be carried out as a group at the beginning and conclusion of each session, respectively.

**Table 1:** Contribution of each component to each training session

	**Time (min)**	**Proportion of session ( *% *)**
Welcome and warming up	5	8
Balance exercises in the form of group training	45	75
Relaxation exercises and closing	10	17
*Total*	60	100

### Experimental group

**Figure 2:**
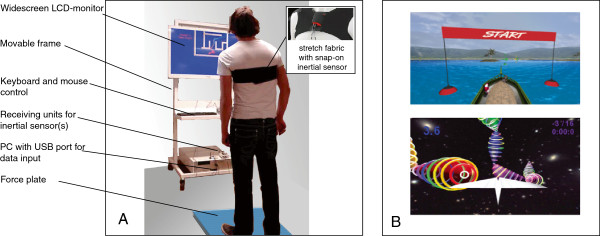
**Illustration of the intervention of the experimental group. A**: Setup of mobile workstation with force plate and/or inertial sensor. **B**: Screenshots of examples of balance games. See text for further details.

In the experimental group VF will be integrated explicitly in each exercise. Three workstations (Motek Medical, Amsterdam, The Netherlands) will be set up in a gym. Figure 2 illustrates the key features of these workstations. They consist of a conventional PC with a 42" flat-panel LCD monitor and movement registration hardware. The latter will be used to map body movements to movements of an object (‘avatar’) on the monitor, by which patients can interact with balance games that are run on the workstations. Six different games will be used, each creating different challenges for the patient within a different virtual environment. Four games focus on controlling body position in space, one on stepping movement, and one on performing a sit-to-stand transfer movement. More accurate and/or faster control of the avatar results in accrual of points.

Movement registration will be accomplished using inertial sensors (Xsens, Enschede, The Netherlands) and a force plate (ForceLink, Culemborg, The Netherlands). Inertial sensors are attached to the patient’s chest using neoprene bands with a snap-on system, providing data about acceleration and rotation of the upper body. Monitoring the sit-to-stand transfer also requires an inertial sensor at the upper leg. The sensors can be easily transferred between patients throughout the training session. The force platform is used to obtain data about center-of-pressure displacements (COP).

The user interface on the workstations is deliberately kept simple and can be operated by a conventional mouse. Patients will receive detailed instructions about the operation of the software including the adjustment of game difficulty. They are encouraged to operate the workstations as autonomously as possible.

### Control group

Participants in the control group will receive equally-dosed balance exercises according to current guidelines for physical therapy in PD [9].

### Outcome measures

#### Descriptors

The MMSE, Hoehn and Yahr stage, disease duration, medication prescription and intake, and weight, length and leg length will be recorded during the first assessment.

#### Primary outcome measure

The Functional Reach Test (FRT) is used as primary outcome measure. The FRT measures the anterior limits of stability as perceived by the subject by assessing the maximal forward reaching distance. This outcome can be classified on the level of *activities* within the international classification of functioning, disability and health [25]. Lim and colleagues reported a mean score of 33.5 ± 7.4 cm in 26 patients with PD (range 22-50 cm) [14]. We note that for patients with PD the minimal detectable change is estimated to be between 9 [26] and 11.5 cm (smallest detectable difference) [27]. The test shows moderate inter- and intra-observer reliability [27,28].

#### Secondary outcome measures

To explore other effects related to training with augmented VF, secondary outcome measures related to balance and gait, the patients’ health status and their level of activity and participation, as well as measures derived from posturographic and EEG measurements will be assessed.

a) *Measures of balance and gait*: the Berg Balance scale assesses how well each of fourteen different balance-related tasks is executed [29]. The timed single leg test is a measure of how long the subject can maintain balance while standing on one leg [30]. The 10 m Walk Test will be used to record walking capacity at comfortable walking speed [26].

b) *Measures of patients’ health status and level of activity and participation*: parts I, II, III and IV of the Unified Parkinson Disease Rating Scale (UPDRS) will be assessed to evaluate disease severity and disease-specific impairments [31]. Items 13–15 and 29–30 of the UPDRS will be used to compute the posture and gait score subscore [15]. The Falls Efficacy Scale consists of ten questions related to the patient’s confidence not to fall during a number of everyday tasks [9,32]. The Parkinson’s Disease Questionnaire (PDQ-39) is a comprehensive questionnaire that provides a measure of health status along eight dimensions [33]. The Hospital Anxiety and Depression Scale addresses feelings of anxiety and depression related to being physically ill [34]. The Multidimensional Fatigue Inventory is a questionnaire developed to obtain insight into the level of fatigue [35].

c) *Posturographic outcome measures*: Tasks during posturographic evaluations consist of quiet stance and rhythmic tracking. Time series of the COP along anterio-posterior and mediolateral axes will be used to compute quantitative measures of task performance. In addition, stabilogram-analysis will be performed to investigate the dynamical and correlative structure of the data [36,37]. For quiet stance we will determine the COP’s variance, its temporal counterpart, the diffusion coefficient, as well as the scaling of the temporal correlation structure (the Hurst exponent). For rhythmic tracking we will follow the same approach but instead of the mere COP trajectory we will employ the distance between (the periodically moving) visual target and the COP in mediolateral direction (as displayed on the monitor). To specifically investigate effects on motor timing, we will also estimate the instantaneous relative phase between target and COP as well as the associated circular variance.

d) *EEG-related outcomes*: EEG will be recorded during the postural task. After artifact-removal based on independent component analysis [20], time-dependent EEG signals will undergo a conventional time-frequency analysis, followed by principal component analysis to identify significant changes in (frequency-dependent) motor-related potentials. We will further determine functional connectivity through neural synchronization in distinct frequency bands, i.e., the variance of the pair-wise relative phase between EEG signals. To localize the sources of brain activity we will combine the EEG signals with the co-registered anatomical MRIs and apply linearly constrained minimum variance beamformers [38,39]. Activities at the sources will finally be analyzed in the same form as the EEG signals at electrode level.

### Measurements and procedures

All assessment sessions will take place in a motion laboratory at VU University Amsterdam, Faculty of Human Movement Sciences. During each session the clinimetric assessments will be performed first, followed by combined posturographic and EEG recordings. An assistant will stand by at all times to prevent falls in the case of a loss of balance.

The clinimetric test battery has been tested extensively in a previous study [27]. The tests will be carried out in accordance with current guidelines [9,40].

**Figure 3:**
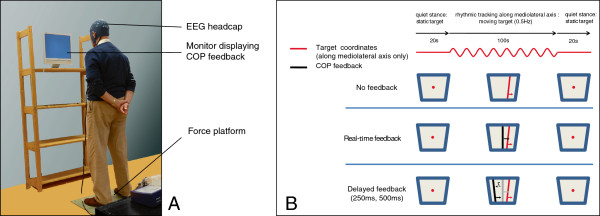
**Illustration of measurement protocol for combined posturography and EEG recordings. A**: Setup of force platform and visual feedback. **B**: Experimental protocol. Each trial consists of a sequence of 20 s quiet stance, 100 s rhythmic tracking, and 20 s quiet stance. During quiet stance only a stationary target is shown. The rhythmic task will be carried out under four different visual feedback conditions: no feedback, real-time feedback, delayed feedback at 250 ms and delayed feedback at 500 ms. See text for further details.

An illustration of the combined posturographic and EEG recordings is provided in Figure 3. The patient will be asked to stand on a 600 mm × 400 mm force plate embedded in the floor (Kistler 9281B; Kistler, Ostfildern, Germany). A monitor providing feedback of the COP position is positioned at eye-height at a distance of ~75 cm from the subject. After familiarization with the setup the patient will perform twelve trials (4 conditions × 3 repetitions), with the possibility to rest after each trial. Each trial will consist of a sequence of 20 s quiet stance, 100 s rhythmic tracking, and 20 s quiet stance. During the rhythmic tracking segment a target will oscillate horizontally on the monitor at a frequency of 0.5 Hz. The patient is asked to match the target as accurately as possible by making whole-body swaying motions from side to side. VF of the COP will be provided by means of a black bar on the monitor. The experimental conditions during the swaying task will include conditions in which VF is withheld, is presented real-time, or is presented with a finite, constant delay of 250 or 500 ms (see Figure 3). From previous studies it is known that VF which is not provided to the subject directly (i.e. real-time) but with a finite delay, may destabilize performance on (tracking) tasks [41-43]. In that case, the better a subject is able to *decouple* from the VF (i.e., the less the subject relies/depends on visual information), the better he/she is able to perform the task. It should be noted that both the task and the VF during assessments will differ from what the patients experience during the training sessions.

During the postural tasks, 64-channel EEG recordings will be collected from Ag/AgCl electrodes that are placed on the scalp by means of a nylon head cap (TMSi, Enschede, The Netherlands). All signals will be high-pass filtered at 0.1 Hz and low-pass filtered at 100 Hz before being sampled at a rate of 2048 Hz (REFA amplifier, TMSi, Enschede, The Netherlands).

Anatomical MRI scans of the brain will be used to substantiate off-line source localization of the EEG signals. If a patient’s scan is not available, a new scan will be taken at VUmc, Department of Radiology.

### Sample size calculation

We calculated the sample size required to obtain sufficient power for finding a difference of 9 cm on the FRT. Pooled estimate of the common variance, calculated from data by Ashburn [44], was entered in a formula for two independent groups with paired observations [45]. A total sample size of twenty-four patients is necessary to find a difference between groups with at least 80% power. To account for smaller effect sizes and an estimated attrition rate of 10%, we plan to include thirty-six patients in total, eighteen in each group.

### Statistical analysis

Group differences in baseline patient characteristics will be tested using χ^2^-tests for categorical data and *t*-tests for continuous variables. Continuous outcome measures that are measured at all three time points will be tested for normality using the Shapiro-Wilk test. If assumptions for parametric testing are met, a mixed design ANOVA with between-subjects factor *group* (experimental vs. control) and within-subjects factor *time* (T0, T1, and T2) will be used to analyze the differential effect of the two interventions and the effect of time. If normality is rejected, or if outcomes are measured on the ordinal level, change scores between T1 – T0 and between T2 – T1 will be subjected to nonparametric Mann–Whitney U-tests. P < 0.05 will be set (two-tailed) as threshold for significance. Relative phase will be assessed through circular statistics using, in particular, Rayleigh tests to analyze group differences of relative phase uniformity between sources [46]. Correlation coefficients will be compared to investigate the association between outcomes.

Statistical analyses regarding EEG outcomes will be conducted using the methods outlined by Houweling and co-workers [47], with adaptations to EEG data regarding the accompanying lead field. Sources will be determined via pseudo *z*-scores of the beamformers that will be tested for within-group consistency through permutation tests [48]. The (relative) phases of the source projected EEG activity in the beta frequency band will be assessed through circular statistics (see above).

## Discussion

The present study investigates the effects of virtual-reality-based visual feedback training in patients with PD not only on behavioral and clinical but also on neurophysiological levels. We hypothesize that balance training with VF may form a valuable contribution to improvements in standing balance.

Balance-training protocols applying augmented VF have been tested clinically in patients with PD [17,18,49]. The results indicate that balance therapy that incorporates some computer-based exercises can be at least as effective as conventional therapy [18,49]. Such VF-assisted balance training could be a cheap alternative to supervised one-on-one therapy, feasible to carry out at home and offering patients extra incentives for training as the exercises incorporate elements of gaming and competition by means of scores. Yet, no studies to date have compared the benefits of conventional balance exercises with exercises that exclusively make use of VF of postural correlates.

### Posturography

Posturographic analyses of balance using a setup of force platform with VF enable the quantification of postural performance and allow for a detailed investigation of its dynamical structure. In the past, researchers have characterized postural sway as chaotic [50,51], purely stochastic [52], or fractional stochastic [36,53-55]. Analyses that quantify the non-linear and stochastic temporal evolution of postural sway have been used to assess the effects of health status and rehabilitation in a number of clinical studies [56-58]. In PD it has been found that postural control mechanisms are characterized by an increase in random fluctuations (short- and long-term diffusion coefficients) in the mediolateral direction [37]. These measures are associated with a history of falls and poor performance on clinical measures of balance. Due to its quantitative and unbiased nature, stabilogram-analysis will enable us to test specific hypotheses involving outcome measures that may be much more sensitive to changes in the control exerted during the postural tasks. Combining the VF setup with a delayed feedback paradigm [41,43] can characterize the extent to which subjects are coupled to the VF. As such we anticipate that these outcomes will form an important adjunct to the clinical outcomes of the proposed RCT.

### Adaptations of functional networks

The positive findings associated with movement strategies that rely explicitly on external stimuli seem to reflect the extent to which the central nervous system capitalizes on the potential to reroute or restructure functional networks. For instance, some of the movement-related neural activity might be rerouted to different brain regions or networks such as the premotor cortex and the cerebellum [12,13,59]. Other researchers have pointed out that internally and externally guided movements may require distinct neural processing [10,11,60-62]. For instance, movements that are externally guided using visual information seem to preferentially activate neurons in the premotor cortex [61], an area which receives visual information directly from the visual cortex and projects directly to the spinal cord (and hence participates in both the visual and the motor network).

Alternatively, it has been suggested that giving explicit feedback or displaying environmental cues may involve attention-related mechanisms [49,63]. This is supported by the finding that some of the benefits of cueing-based therapy have been shown to carry-over to situations without cues [15,16,63,64]. This observation is difficult to explain if it were the availability of cues alone that is instrumental to functional improvement.

The present proof-of-concept trial will certainly help to highlight benefits of a balance-training program based on augmented visual feedback. How improvement is achieved in terms of altered motor control will be determined through the co-registered posturographic recordings. We believe that the complex nature of PD calls for a multimodal assessment approach to unravel the underlying mechanisms that influences the effects of VF on the quality of balance control.

## Abbreviations

COP: Center-of-pressure; EEG: Electroencephalography; FRT: Functional reach test; PD: Parkinson’s disease; RCT: Randomized clinical trial; VF: Visual feedback; VUmc: VU University medical center.

## Competing interests

The authors declare that they have no competing interests.

## Authors’ contributions

EvW and GK obtained funding for the study. MvdH, CdG, IB, PB, AD, GK and EvW contributed to the research design, the intervention, outcome measures and project management (clinimetrics/intervention: EvW, CdG, IB, and MvdH; EEG and posturography: AD, MvdH, and EvW). MvdH is principally responsible for the assessments, data-analysis and drafting of the manuscript. All authors critically reviewed the manuscript and approved the submitted version.

## Pre-publication history

The pre-publication history for this paper can be accessed here:

http://www.biomedcentral.com/1471-2377/13/137/prepub
